# Poster Session I - A79 A PILOT RANDOMIZED CONTROLLED TRIAL TO EVALUATE EFFECT OF LATE VS EARLY INTRODUCTION OF GLUTEN-FREE OATS ON SYMPTOMATIC, SEROLOGIC AND DISEASE ACTIVITY IN PATIENTS WITH NEWLY DIAGNOSED CELIAC DISEASE

**DOI:** 10.1093/jcag/gwaf042.079

**Published:** 2026-02-13

**Authors:** N Chang, J Blom, A Verma, D Borovsky, S Tandon, A Caminero, D Gidrewicz, J Turner, S Case, M Pinto-Sanchez

**Affiliations:** Gastroenterology, McMaster University, Hamilton, ON, Canada; Gastroenterology, McMaster University, Hamilton, ON, Canada; Gastroenterology, McMaster University, Hamilton, ON, Canada; McMaster University Faculty of Health Sciences, Hamilton, ON, Canada; Gastroenterology, McMaster University, Hamilton, ON, Canada; McMaster University Faculty of Health Sciences, Hamilton, ON, Canada; University of Calgary, Calgary, AB, Canada; University of Alberta, Edmonton, AB, Canada; Canadian Celiac Association, Mississauga, ON, Canada; Gastroenterology, McMaster University, Hamilton, ON, Canada

## Abstract

**Background:**

Gluten-free diet (GFD) is the only treatment for celiac disease (CeD), requiring strict avoidance of wheat, rye, and barley. Pure oats are safe for most CeD, though cross-contamination with other sources of gluten is common. Oats are highly nutritious, yet some experts recommend delaying their introduction for six months to minimize symptoms, despite limited evidence and lack of consensus on this approach.

**Aims:**

To evaluate the impact of early versus late introduction of gluten-free oats on symptoms, nutritional status, diet quality, celiac disease activity, and quality of life in recently diagnosed celiac disease patients

**Methods:**

We conducted a pilot RCT with two arms in parallel in biopsy-proven CeD patients within 3 months of diagnosis. The study period was 6 months and involved 3 visits. Patients were randomized into one of two arms: 1) Early introduction of oats (start GF oats immediately after the diagnosis); 2) Late introduction of GF oats (remove oats from diet and introduce GF oats at 6 months). Participants in both arms met a registered dietician for GFD education. They completed questionnaires assessing dietary intake, oats intake, gastrointestinal symptoms (GSRS), Celiac Symptom Index (CSI) for symptoms related to celiac disease, and the Celiac-related quality of life (CeD-QoL) at baseline, 3, and 6 months after. Clinical activity related to CeD was defined based on an established cut-off of > 30 in CSI.

**Results:**

From June 2023 to June 2025, 559 patients visited the McMaster University Medical Centre’s Celiac Clinic, of whom 85 met eligibility criteria. Of them, 37 consented, with 6 dropping out before randomization, and 31 were randomized. Of these, 21 patients completed the study (n = 11 ‘early’ and n = 10 ‘late’ oats), and 10 visits are ongoing. The recruitment rate averaged two patients per month, with retention rates of 67.7%. Preliminary analysis of those who completed the study showed no differences in baseline demographics between groups. After 6 months, there were no differences in the overall celiac-related symptoms between groups (CSI >30 early vs late= 22.5% vs 25.0%). The early oats group reported less gastrointestinal symptoms compared with late oats group [Median GSRS early vs late = 23.0 (11) vs 28.5 (37)]. No differences in QoL scores were observed in those randomized to early oats compared with late oats [Median CeD- QOL early vs late= 44.0 (24) vs 45.5 (49)].

**Conclusions:**

Our preliminary analysis suggests that incorporating gluten-free oats into a gluten-free diet may alleviate gastrointestinal symptoms in celiac patients, challenging current dietary recommendations and highlighting the need for further clinical investigation.

**Funding Agencies:**

Regional Medical Associates (RMA) Scholarship Fund (McMaster University), J. A. Campbell Research Grant (Celiac Canada)

